# The combination of L-4F and simvastatin stimulate cholesterol efflux and related proteins expressions to reduce atherosclerotic lesions in apoE knockout mice

**DOI:** 10.1186/1476-511X-12-180

**Published:** 2013-12-08

**Authors:** Ru Ying, Yong Yuan, Ya-Fei Qin, Di Tian, Li Feng, Zhi-Gang Guo, Yan-Xiang Sun, Ming-Xing Li

**Affiliations:** 1 Department of Cardiology, Zhongshan hospital, Sun Yat- Sen University, Zhongshan, Guang Dong, China; 2 Department of Cardiology, Nanfang Hospital, Southern Medical University, Guangzhou, Guangdong, China

**Keywords:** Atherosclerosis, High density Lipoprotein, Coronary artery disease, Apolipoprotein A-1 mimetic peptide, Statins

## Abstract

**Background:**

Both L-4F, one apolipoprotein A-1 mimetic peptide, and statins can reduce progression of atherosclerosis by different mechanisms. The combination of the two drugs can cause lesion regression by rendering HDL anti-inflammatory. We postulated that combination of L-4F and simvastatin may stimulate cholesterol efflux and related proteins expressions to alleviate atherosclerosis.

**Methods:**

Thirty male wild-type (W-T) C57 BL/6 mice and apo E^−/−^ mice were divided into five groups: W-T group, atherosclerosis (AS) group, simvastatin group, L-4F group and the combination of simvastatin and L-4F group. After 16 weeks, serum lipids, atherosclerotic lesion areas, cholesterol efflux and the expressions of related proteins including ABCA1, SR-BI, ABCG1, LXRα and PPARγ were evaluated.

**Results:**

The aortic atherosclerotic lesion areas were reduced more significantly by combination of both drugs than single agent, and cholesterol efflux was promoted more in combination group than simvastatin and L-4F group. Besides, the combination group promoted expressions of cholesterol efflux related proteins.

**Conclusions:**

The combination of L-4F and simvastatin reduced atherosclerotic lesions, which stimulates cholesterol efflux by promoting the expressions of related proteins. In addition, these results help us further understand that the regression of the atherosclerosis would be assessed by reduction in LDL-C with increase of cholesterol efflux.

## Background

Randomized clinical trials have shown that statins reduce the progression of atherosclerosis (AS) and the incidence of cardiovascular events. Statins are appreciated and accepted by clinical staff as a first-line choice for the treatment of coronary artery disease (CAD) in humans. However, statins can reduce a part of cardiovascular events in spite of lowering effectively and efficiently low density lipoprotein cholesterol (LDL-C). So it is high time to seek potent treatment strategy.

A robust inverse association between the level of high density lipoprotein cholesterol (HDL-C) and the risk of cardiovascular disease has fostered intensive research seeking to target HDL metabolism for therapeutic gain [1,2]. When it is challenging to reduce clinical CAD risk by pharmacologic increases in HDL-C levels, the functions of HDL are increasingly focused on, including the ability to mediate reverse cholesterol transport (RCT), antioxidant and anti-inflammatory capacities, nitric oxide-promoting activity and so on. Although cholesterol efflux from macrophages represents only a small fraction of overall flux through the RCT pathway, it is probably the component that is most relevant to atheroprotection [3]. Amit V. Khera et al. reported that cholesterol efflux capacity from macrophages has a strong inverse association with subclinical atherosclerosis and CAD, independently of the HDL-C level. Cholesterol efflux capacity, as an integrated measure of HDL quantity and quality, is reflective of the role of HDL in atheroprotection [4]. Several lipid transporters have been showed to promote cholesterol efflux in vitro and vivo, and the key ones include ATP Binding Cassette A1 (ABCA1), ATP Binding Cassette G1 (ABCG1) and Scavenger Receptor Class B type I (SR-BI) [5-8]. Moreover, some nuclear receptors play central roles in cholesterol metabolism, such as Liver X receptors (LXRs) [9] and peroxisome proliferater-activated receptors (PPARs) [10]. When activated, they induce a series of genes that are involved in cholesterol efflux.

Apo A-1, the main protein component of HDL, plays an essential role in anti-atherosclerosis. Apolipoprotein A-1 mimetic peptides are made to mimic the amphipathic alpha helix of ApoA-1 [11], and have the similar functions with apoA-1. L-4F, a type of apo A-1mimetic peptides, has a characteristic of high biological activity. Over the last several years, studies have illustrated the capacities of L-4F to imitate many of the protective functions associated with ApoA-1, such as promotion of vasodilation and anti-inflammatory effects [12,13].

Both simvastatin and apo A-1mimetic peptides can reduce atherosclerosis by different mechanisms, and the combination of them can promote anti-inflammatory function of HDL and alleviate atherosclerosis. Nevertheless, it has not been elucidated whether they can stimulate cholesterol efflux and reduce atherosclerotic lesions. Thus, we aimed to investigate the anti-atherogenic effect of the combination of L-4F and simvastatin, and determine the mechanisms including cholesterol efflux and the expressions of related proteins like ABCA1, SR-BI, ABCG1, LXRα and PPARγ.

## Results

### Serum lipids

The results of the serum lipids (Table 1) suggested that hypercholesterolemia model was successfully established. Serum HDL-C levels in simva group were significantly higher and TC, TG and LDL-C concentrations were significantly lower than AS group. In contrast, L-4F group only increased the concentration of apo A-1, but not changed other serum lipids. The combination group increased the HDL-C and apo A-1 levels and decreased TC, TG and LDL-C levels.

**Table 1:** **Serum TC**, **LDL**-**C**, **HDL**-**C**, **TG and Apo A**-**I levels** (**n** = **6**)

	**TC/(mmol/L)**	**TG/(mmol/L)**	**LDL-C/(mmol/L)**	**HDL-C/(mmol/L)**	**Apo A-I/(mg/ml)**
W-T group	3.17 ± 0.53^2^	0.91 ± 0.32^2^	0.55 ± 0.10^2^	2.27 ± 0.32^2^	0.95 ± 0.14^2^
AS group	22.42 ± 1.99	2.68 ± 0.28	12.45 ± 1.29	1.60 ± 0.22	0.73 ± 0.09
Simva group	14.89 ± 2.06^2^	2.34 ± 0.17^1^	7.59 ± 1.25^2^	2.03 ± 0.40^1^	0.86 ± 0.10
L-4F group	20.83 ± 2.11	2.58 ± 0.27	11.75 ± 0.92	1.73 ± 0.23	0.89 ± 0.09^1^
Simva + L-4F	13.60 ± 2.50^2^	2.25 ± 0.29^2^	7.20 ± 0.68^2^	1.99 ± 0.20^2^	1.06 ± 0.16^2*^

^1^*P* < 0.05, ^2^*P* < 0.001, vs. AS group; ^a^*P* < 0.05, ^b^*P* < 0.001, vs. Simva group.

*P < 0.05, **P < 0.001, vs. L-4F group.

*TC* (total cholesterol), *TG* (Triglycerides), *LDL*-C (low density lipoprotein cholesterol), *HDL*-*C* (high density lipoprotein cholesterol).

### AS lesion areas

**Figure 1:**
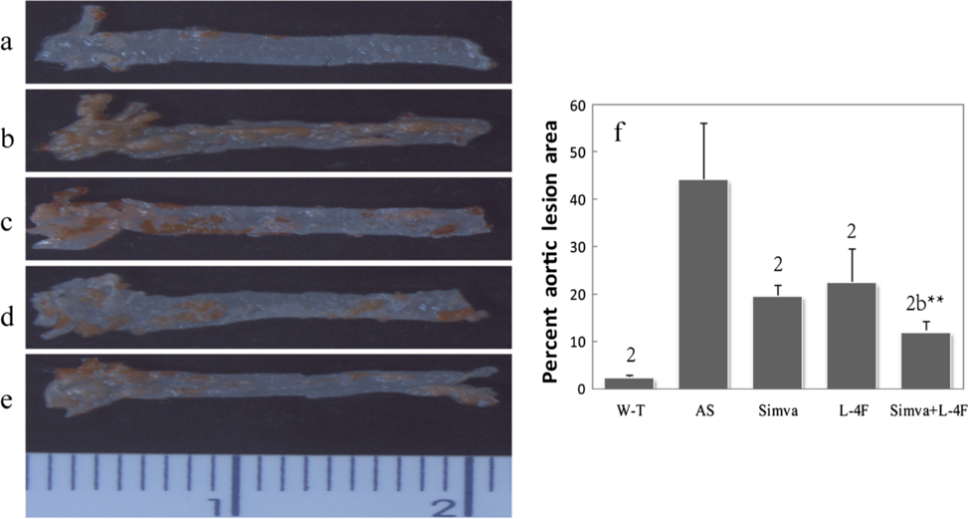
**Atherosclerotic lesion areas in ascending and thoracic aorta stained with Oil Red O (n = 6). a-e** the aorta stained with Oil Red O in five groups, **a**: W-T group; **b**: AS group; **c**: Simva group; **d**: L-4F group; **e**: Simva + L-4F group. L-4F was administered intraperitoneally daily at a dose of 1 mg/kg/d, simvastatin intragastrically at a dose of 5 mg/kg/d and the combination group was given L-4F 1 mg/kg/d and simvastatin 5 mg/kg/d. **f** Lesion area was expressed as a percentage of total area. Simvastatin and L-4F equally reduced the lesion areas, and when they used together the lesion areas were the least.^1^*P* < 0.05, ^2^*P* < 0.001, vs. AS group; ^a^*P* < 0.05, ^b^*P* < 0.001, vs. Simva group;^*^P < 0.05, ^**^P < 0.001, vs. L-4F group.

The percentage plaque area was significantly higher in the AS group (44.30 ± 11.85%) than W-T group (2.54 ± 0.25%), which indicated that the expected atherosclerotic model were successful. Additionally, aortic lesion area was significantly reduced in all the treated groups (19.77 ± 1.95%, 22.58 ± 6.88% and 12.17 ± 1.90%) compared with the AS group (Figure 1).

### Cholesterol efflux capacity

**Figure 2:**
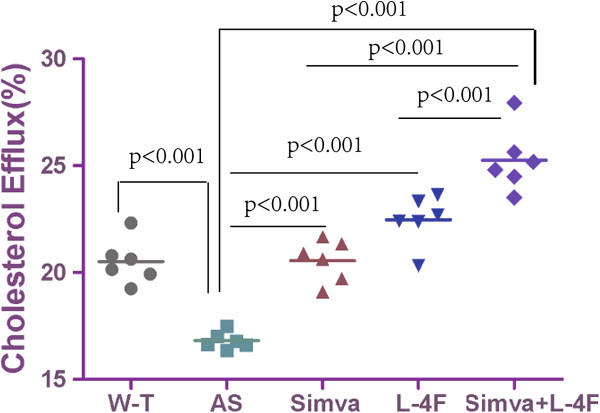
**Cholesterol efflux capacity from RAW264.7 cells.** The high-fat diet significantly reduced the cholesterol efflux, and L-4F and simvastatin both improved cholesterol efflux.

Compared with the W-T group (20.51 ± 1.04%), the cholesterol efflux of atherosclerotic mice was impaired (16.82 ± 0.39%). Simvastatin (20.55 ± 0.98%) and L-4F (22.47 ± 1.16%) significantly increased cholesterol efflux capacity. The combination group of simvastatin and L-4F (25.26 ± 1.50%) promoted cholesterol efflux most effectively (Figure 2).

### mRNA expressions of ABCA1, SR-BI, ABCG1, LXRα and PPARγ in liver and macrophage

**Figure 3:**
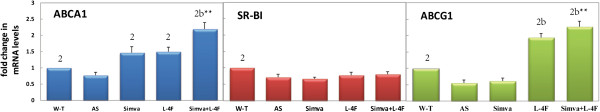
**Fold change in mRNA levels of ABCA1, SR**-**BI and ABCG1 in macrophage by RT-PCR.** Simvastatin and L-4F could not change the expression of SR-BI in macrophages. L-4F increased the mRNA expressions ABCA1 and ABCG1, and simvastatin improved the expression of ABCA1. ^1^*P* < 0.05, ^2^*P* < 0.001, vs. AS group; ^a^*P* < 0.05, ^b^*P* < 0.001, vs. Simva group;^*^P < 0.05, ^**^P < 0.001, vs. L-4F group.

In liver, simvastatin increased the mRNA expressions of ABCA1, ABCG1, LXRα and PPARγ; L-4F and the combination group promoted the expressions of ABCA1, SR-BI, ABCG1, LXRα and PPARγ (Table 2). In macrophage, we just detected the expressions of ABCA1, SR-BI and ABCG1 because of the limited cells. The results suggested that simvastatin just increased the expression of ABCA1 and L-4F and the combination group increased the expressions of ABCA1 and ABCG1 (Figure 3).

**Table 2:** **mRNA expressions of ABCA1**, **SR**-**BI**, **ABCG1**, **LXRα and PPARγ in liver**

	**ABCA1**	**SR**-**BI**	**ABCG1**	**LXRα**	**PPARγ**
W-T group	1^2^	1^2^	1^2^	1^2^	1^2^
AS group	0.52 ± 0.08	0.64 ± 0.06	0.75 ± 0.12	0.40 ± 0.07	0.50 ± 0.08
Simva group	0.98 ± 0.14 ^2^	0.69 ± 0.11	1.23 ± 0.19^2^	0.71 ± 0.07^2^	0.96 ± 0.07^2^
L-4F group	1.38 ± 0.12 ^2b^	1.01 ± 0.14^2b^	1.46 ± 0.16^2b^	1.08 ± 0.10^2b^	1.19 ± 0.20^2b^
Simva + L-4F	1.51 ± 0.13 ^2b^	1.96 ± 0.49^2**^	1.73 ± 0.24^2b**^	1.31 ± 0.20^2b**^	1.52 ± 0.10^2b**^

^1^*P* < 0.05, ^2^*P* < 0.001, vs. *AS* group; ^a^*P* < 0.05, ^b^*P* < 0.001, vs. Simva group.

^*^P < 0.05, ^**^P < 0.001, vs. L-4F group.

### Protein expressions of ABCA1, SR-BI and ABCG1 in liver and macrophage

**Figure 4:**
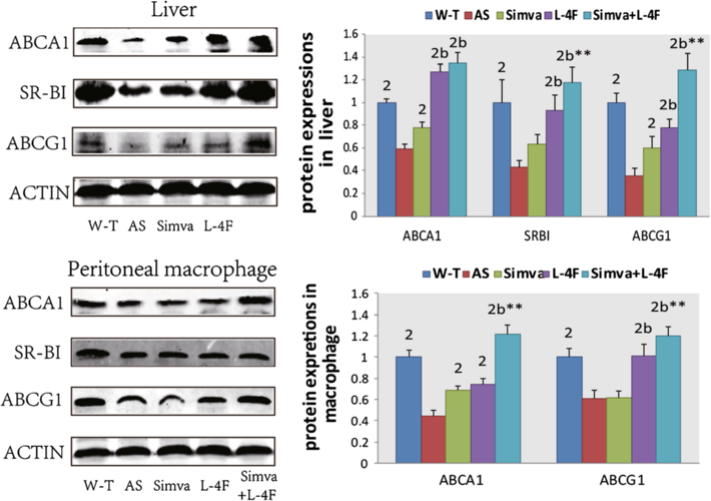
**Protein expressions of ABCA1, SR-BI and ABCG1 in liver and macrophage by Western blot.** Simvastatin increased the expressions of ABCA1 and ABCG1 in liver and ABCA1 in macrophage, Both L-4F and the combination group improved the expressions of ABCA1, SR-BI and ABCG1 in liver and ABCA1 and ABCG1 in macrophage. ^1^*P* < 0.05, ^2^*P* < 0.001, vs. AS group; ^a^*P* < 0.05, ^b^*P* < 0.001, vs. Simva group;^*^P < 0.05, ^**^P < 0.001, vs. L-4F group.

In liver, simvastatin increased the protein expressions of ABCA1 and ABCG1; L-4F and the combination group promoted the expressions of ABCA1, SR-BI and ABCG1. In macrophage, the expression of SR-BI in all the three treated groups was not altered. Simvastatin increased the expression of ABCA1 and L-4F and the combination group elevated the expressions of ABCA1 and ABCG1 (Figure 4).

### Protein expressions of ABCA1, SR-BI, ABCG1, LXRα and PPARγ in abdominal aorta

**Figure 5:**
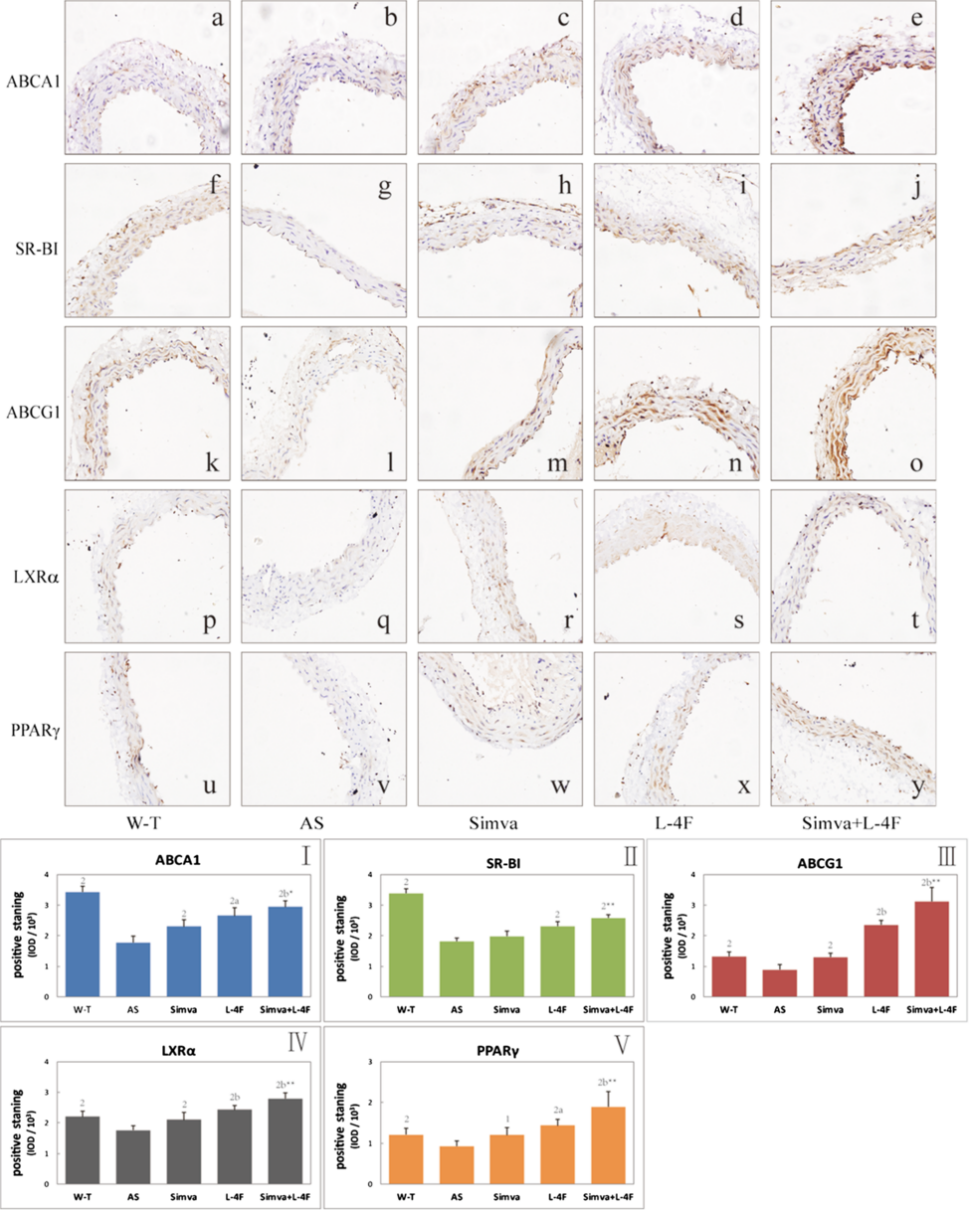
**Immunochemical staining of ABCA1**, **SR**-**BI**, **ABCG1**, **LXRα and PPARγ in abdominal aortic. a-y** representative light microscopy images of abdominal aorta (X400). **a-e** ABCA1 staining; **f-j** SR-BI; **k-o** ABCG1; **p-t** LXRα; **u-y** PPARγ.I-V statistical results of positive staining (n = 6). ^1^*P* < 0.05, ^2^*P* < 0.001, vs. AS group; ^a^*P* < 0.05, ^b^*P* < 0.001, vs. Simva group;^*^P < 0.05, ^**^P < 0.001, vs. L-4F group.

There are expressions of ABCA1, SR-BI, ABCG1, LXRα and PPARγ in abdominal aorta in mice. Simvastatin increased the expressions of ABCA1, ABCG1, LXRα and PPARγ in aorta. L-4F and the combination group promoted the expressions of ABCA1, SR-BI, ABCG1, LXRα and PPARγ (Figure 5).

### Correlation analysis

Both cholesterol efflux (r = 0.579, *P* < 0.001) and HDL-C (r = 0.568, *P* < 0.001) were inversely correlated with AS lesion area by Pearson correlation analysis.

## Discussion

Reverse cholesterol transport (RCT) is considered to be important in preventing the buildup of lipids that predisposes to atherosclerosis [14]. The process of RCT can be divided into three stages [15]. RCT begins with cellular cholesterol efflux to HDL from peripheral cells; Subsequently, the cholesterol in HDL in blood is transported to the liver. At last, the cholesterol is delivered to hepatocytes from HDL, and excreted through the bile. Although cholesterol efflux from macrophages is only the initial stage of the RCT, it has emerged as a biomarker of RCT. In this study, we confirmed that cholesterol efflux was inversely associated with the lesion of AS and we also believed that cholesterol efflux could serve as a stronger predictor of AS and coronary disease status even than serum HDL-C in different populations including normal or lipid disordered or drug-treated people. The results of this study help us further understand that the regression of the atherosclerotic plaque would be observed by reduction in LDL-C together with increase of cholesterol efflux.

Statins are largely used in patients with atherosclerosis, and lower the rate of cardiovascular death. The majority of trials with statins investigated patients with CAD, in whom the reduction in cardiovascular events appeared to be related to the cholesterol-lowering effect of statins and ultimately to plaque stabilization [16]. However, emerging evidence suggests that statins do more than act as inhibitors of cholesterol biosynthesis. Many other actions have been attributed to statins including anti-inflammatory, anti-oxidative, anti-proliferative and antithrombotic effects and so on [17]. In this study, we found that simvastatin could promote cholesterol efflux, which might be one of the mechanisms of anti-atherosclerosis of simvastatin. It increased the expressions of ABCA1, ABCG1, LXRα and PPARγ in liver and aorta and ABCA1 in macrophage that probably facilitated the cholesterol efflux. Several reports showed statins enhances the cholesterol efflux capacity [18,19], and simvastatin promoted the expression of hepatic genes associated with reverse cholesterol transport [20]. However, no increase was noted after patients had been treated with pravastatin and atorvastatin [4]. We assumed that the discrepancy might be partly attributable to different models.

The beneficial effects of HDL on atherosclerosis have greatly been attributed to its major protein, apoA-I. 4 F has the similar functions to apo A-I and high biological activity, including L-4F and D-4 F. They have the similar functions such as reduction of lesion formation and anti-inflammation, but L-4F is not orally efficacious because of digestion of L-4F by gut proteases. D-4 F was reported to stimulate cholesterol efflux [21], however it is not determined whether L-4F has the same effect or not. Our finding indicated L-4F improves cholesterol efflux capacity. Moreover, in this experiment, we observed for the first time that L-4F had the atheroprotective effect in parallel with simvastatin in mice. Though, of course, its effects on humans need further research.

**Figure 6:**
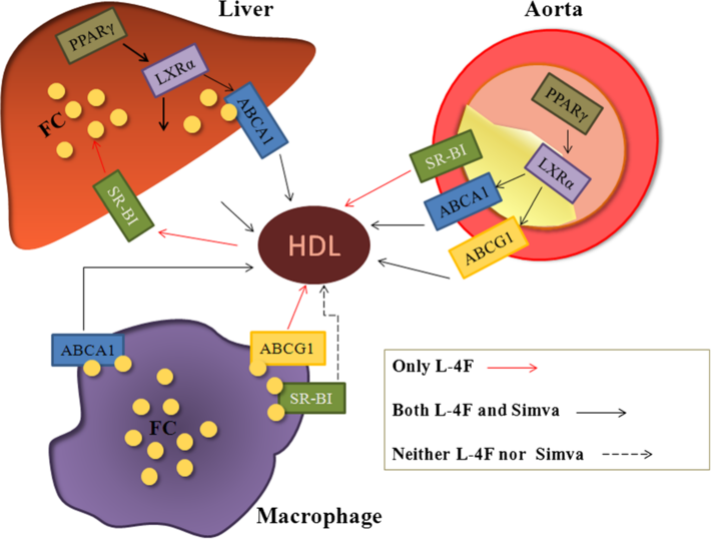
**Deduced mechanisms by which L-4F and simvastatin promote reverse cholesterol transportation (RCT).** The red arrows mean only L-4F increases the expressions of proteins, and black arrows mean both L-4F and simvastatin up-regulate the expressions of proteins. Neither L-4F nor simvastatin promote the expressions of proteins when it is fine dotted line.

There are multiple pathways by which excess cholesterol from peripheral tissue or cells can be removed by HDL including ABCA1, ABCG1 and SR-BI. One important pathway for cholesterol-mediated efflux involves interaction between ABCA1 and cholesterol-deficient and phospholipid-depleted apo A-I complexes [22-24]. In our research, we observed that both L-4F and simvastatin up-regulated the expressions of ABCA1 in macrophages, livers and aortic walls (Figure 6). That indicated that they accelerated cholesterol removal from macrophages and plaques and facilitated biliary excretion of cholesterol in livers. Subsequently, ABCG1 mediates macrophage cholesterol efflux through interactions with spherical, cholesterol -containing HDL particles, medium HDL, large HDL, and very large HDL [6,25]. Our results inferred that the expression of ABCG1 in macrophages, livers and aortic walls were up-regulated by L-4F, and simvastatin increased the expression of ABCG1 in livers and aortic walls, not macrophages. ABCG1 might be expected to be inhibitory of atherosclerosis development by promoting macrophage efflux. In contrast, SR-BI is a multifunctional receptor that mediates bidirectional lipid transport in the macrophage, which is dependent on the content of cholesterol in lipid-laden macrophages. The present study suggested that L-4F up-regulated the expression of SRBI in livers and aortic walls but not macrophages, and simvastatin did not alter the expression of SR-BI. SR-BI is known to promote cholesterol efflux from macrophage to HDL as an acceptor [26], but this flux can be bidirectional and has uncertain effects on macrophage cholesterol mass which accounted for failing to up-regulate the expression of SRBI in macrophages in our research.

At present, clinical staff shows a strong interest in ligands for LXR_S_ and PPARs for the treatment of cardiovascular disease, and they are ligand-activated transcription factors that plays well-established roles in up-regulating transcription of ABCA1 and ABCG1 [27,28]. Our current study suggested that L-4F and simvastatin up-regulated the expression of LXRα and PPARγ in livers and aorta. Several studies have shown that synthetic LXR agonists induce cholesterol efflux and reverse cholesterol transport by up-regulating ABCA1 and ABCG1 expression [29,30]. LXR agonists also improve glucose metabolism, antagonize inflammatory responses, inhibit vascular smooth muscle cell proliferation and protect against the development of atherosclerosis in mice [31]. PPARs regulate diverse aspects of lipid and glucose metabolism. Agonists for the PPAR have also been reported to increase ABCA1 and ABCG1 expression through up-regulating LXR expression [28].

D-4 F and Statins has been previously showed to cause lesion regression by render HDL anti-inflammatory [32]. This is the first report of regression of atherosclerotic lesions in mice administered L-4F and simvastatin by an apparent activation of cholesterol efflux. That means the combination of a statin and apolipoprotein A-1 mimetic peptide can alleviate atherosclerosis by increasing the functions of HDL like anti-inflammatory and cholesterol efflux.

## Conclusions

In summary, our results showed that the combination of L-4F and simvastatin can alleviate atherosclerosis more significantly than given as single agents in mice, which stimulates cholesterol efflux by promoting the expressions of related proteins. Thus, we put forward that the combination of simvastatin and L-4F may be a particularly potent treatment strategy.

### Limitations

There are limitations in extrapolating results from murine models to humans. Mice are relatively resistant to statins [33] but respond well to 4 F [34], which may account for the paralleled anti-atherosclerotic effect of L-4F and simvastatin in mice. Future studies will be required to determine whether the combination of L-4F and simvastatin produces results in humans similar to the present results here for mice and if so, which regimen will be superior.

L-4F and simvastatin were showed to have the similar anti-atherosclerotic effect, but the conclusion was limited due to the absence of a dose–response experiment to precisely prove it. However, we used the regular dosage of each drug to compare their effects with each other which we thought was convincing.

Our research is teamwork. In other parts of our experiments, we observed that the combination of L-4F and simvastatin significantly increased paraoxonase-1 activity, rendered HDL anti-inflammatory, decreased Dil-Ox-LDL influx from macrophages and reduced the concentrations of cholesterol and cholesteryl ester in macrophages. All of them with the results presented here may have more complete explanations for the effects of the combination of both drugs.

## Methods

### Animals

Thirty 8-week-old male wild-type C57 BL/6 and apo E^−/−^ mice (C57 BL/6 background), purchased from Southern Medical University institute of animals, were randomly divided into five groups (n = 6/group) after acclimatization for several weeks, (1) wild type (W-T) group (C57 BL/6 mice receiving vehicle and normal diet), (2) atherosclerosis (AS) group (apo E^−/−^ mice receiving vehicle and high-fat diet), (3) simvastatin (Simva) group (apo E^−/−^ mice receiving high-fat diet and simvastatin), (4)L-4F group (apo E^−/−^ mice receiving high-fat diet and L-4F) and (5) the combination (Simva + L-4F) group (apo E^−/−^ mice receiving high-fat diet and simvastatin and L-4F). All animal studies were performed using the protocols approved by the Institutional Animal Care and Use Committee of Southern Medical University at Guangzhou. L-4F was administered intraperitoneally daily at a dose of 1 mg/kg/d [12]. Peptides L-4F contained 18 amino acids with the sequence DWFKAFYDKVAEKFKEAF.

### Serum lipids analysis

Blood was collected by cardiac puncture under anesthesia of diethyl ether after 8-h fast. Triglycerides (TG), total cholesterol (TC), HDL-C and LDL-C were measured by using an automated biochemical analyzer. Apo A-1 concentrations were determined by ELISA kit.

### Isolation of peritoneal macrophages

Four days after intraperitoneal thioglycollate injection, peritoneal macrophages were harvested from the experimental mice. Following collecting, peritoneal macrophages were immediately frozen at −80°C until analysis.

### Lesion quantification

After the blood, peritoneal macrophage and liver samples were taken, the aorta was cut into two sections separately prepared for quantification of aortic lesion area and immunohistochemistry. The ascending aorta and thoracic aorta were then stained with Oil Red O and lesions were quantified by video capture under a stereo dissecting microscope. Lesion and total areas were determined using Image-Pro Plus software 6.0.

### Cholesterol efflux analysis

Experiments were performed as previously described [4]. RAW264.7 cells, derived from a murine macrophage cell line, were plated in DMEM medium supplemented with 10% fetal bovine serum and radiolabeled with 30 μg /ml acetylated-LDL and 1 μCi/ml ^3^H-cholesterol (Perkin-Elmer) for 24 h, and incubated with 0.3 mM 8-Br-cAMP for 6 hours. Thereafter, cells were washed again and equilibrated in DMEM medium supplemented with 2% bovine serum albumin for 18 h. Subsequently, efflux mediums containing 2% mice serum were added for 4 hours. Liquid scintillation counting was used to quantify the efflux of radioactive cholesterol from the cells. Percent efflux was calculated by the following formula: [(microcuries of ^3^H-cholesterol in mediums containing mice serum − microcuries of ^3^H-cholesterol in serum-free mediums) ÷ microcuries of ^3^H-cholesterol in cells extracted before the efflux step] × 100.

### Quantitative real-time PCR

Total RNA from mice livers and macrophages was extracted by TRIZOL Reagent (Invitrogen). cDNA synthesis was performed from 1ug of total RNA using reagents from TOYOBO. Real-time PCR was performed using a SYBR-green PCR master mix kit (Takara). Real-time quantitative PCR was carried out on an ABI-Prism 7500 (BioRad, American) sequence detector with the defaults settings. The primers, designed with the Primer Express Software (Applied Biosystems) and synthesized by Invitrogen Ltd, were listed in Table 3. PCRs for each sample were performed in duplicate. Relative mRNA levels were calculated by the method of 2^-△△Ct^.

**Table 3:** **Primers used for real**-**time PCR analysis**

**Gene**	**Primer**	**Sequence (5′-3′)**
ABCA1	Sense	5′- CTT CCC ACA TTT TTG CCT GG–3′
	Anti-sense	5′- AAG GTT CCG TCC TAC CAA GTC C–3′
SR-BI	Sense	5′- GCA AAT TTG GCC TGT TTG TT–3′
	Anti-sense	5′- GAT CTT GCT GAG TCC GTT CC–3′
ABCG1	Sense	5′- CGA GAG GGC ATG TGT GAC G–3′
	Anti-sense	5′- CCG AGA AGC TAT GGC AAC C–3′
LXRα	Sense	5′- TAG GGA TAG GGT TGG AGT CAG–3′
	Anti-sense	5′- AGT TTC TTC AAG CGG ATC TGT–3′
PPARγ	Sense	5′- ATA AAG CAT CAG GCT TCC ACT–3′
	Anti-sense	5′- GCA CTT CTG AAA CCG ACA GTA–3′
β-actin	Sense	5′-CAG ATC ATG TTT GAG ACC TTC AAC–3′
	Anti-sense	5′- TCG AAG TCT AGA GCA ACA TAG CAC–3′

*ABCA*1 (ATP Binding Cassette A1); *SR*-*BI* (Scavenger Receptor Class B type I); *ABCG1* (ATP Binding Cassette G1); *LXRα* (Liver X receptor α); *PPARγ *(peroxisome proliferater-activated receptor γ).

### Western blots

Tissues and cells were harvested and protein extracts prepared. They were then subjected to Western blot analyses (10% SDS-PAGE; 25 μg per lane) using anti-ABCA1, SR-BI (Novus), ABCG1, LXRα and PPARγ (Abcam Co.Ltd, American) and β-actin antibodies. The proteins were visualized and quantified using the Image J analysis software program.

### Abdominal aortic immunohistochemistry

All segments were embeded in paraffin and cut into 4-μm cross sections for histological examination. Experiments were performed as previously described [35]. Immunostaining for ABCA1, SR-BI, ABCG1, LXRα and PPARγ was performed in paraffin-embedded. Antibody binding was visualized with SABC kits (Boster Biotechnology Co. Ltd), Diaminobenzidine (DAB) was used as the nuclear counterstain. All sections for microscopic quantification were captured under an Olympus BX51 light microscope equipped with a DP70 digital camera (Olympus, Tokyo, Japan) and were measured with Image Proplus 6.0 image analysis software.

### Statistics

Data are expressed as means ± SE. Differences between groups were evaluated using the SPSS 13.0. Groups were compared by one-way analysis of variance (ANOVA). Post-hoc comparisons were made among the various groups using least significant difference (LSD) method when variances are not homogeneity, and Dunnett’s T3 method when variances are homogeneity. Coefficients of correlation (r) were calculated by Pearson correlation analysis. A probability value of *P* < 0.05 was considered to be significant.

## Abbreviations

AS: Atherosclerosis; CAD: Coronary artery disease; LDL-C: Low density lipoprotein cholesterol; HDL-C: High density lipoprotein cholesterol; RCT: Reverse cholesterol transport; ABCA1: ATP binding cassette A1; ABCG1: ATP binding cassette G1; SR-BI: Scavenger receptor class B type I; LXRs: Liver X receptors; PPARs: Peroxisome proliferater-activated receptors; TG: Triglycerides; TC: *Total cholesterol*.

## Competing interests

The authors declare that they have no competing interests.

## Authors’ contributions

YY was responsible for the experimental design, supervising the project, data analysis and revising the manuscript. RY carried out all aspects of experiments, data analysis and drafted the manuscript. YFQ was involved in lesion quantification and abdominal aortic immunohistochemistry, and all samples collected. DT participated in constructed animal models. LF and ZGG were involved in supervising the project. YXS and MXL revised the manuscript. All authors read and approved the final manuscript.
